# Patients' auto report of regularity of their menstrual cycles. Medical history is very reliable to predict ovulation. A cross-sectional study

**DOI:** 10.5935/1518-0557.20160027

**Published:** 2016

**Authors:** Reinaldo S. A. Sasaki, Mario S. Approbato, Mônica C. S. Maia, Eliamar Aparecida de B. Fleury, Christiane R. Giviziez, Neuma Zanluchi

**Affiliations:** 1 Human Reproduction Center - Department of Obstetrics and Gynecology, Clinical Hospital - Goias Federal University, Goiânia/GO, Brazil; 2 EMAC- Goias Federal University - Goiânia/GO, Brazil; 3 Goias Federal University - Jataí/GO, Brazil; 4 Hospital de Doenças Tropicais - Goiânia/GO, Brazil

**Keywords:** Infertility, Menstrual Cycle, Ovulation Detection, Ultrasonography

## Abstract

**Objective:**

Infertility of ovulatory cause can account for a quarter of infertility etiologies and one of the questions in the patients' clinical history is about their self-perception of the regularity of their menstrual cycles. The aim of this study was to assess whether the information on menstrual regularity is consistent with the assessment of the presence or absence of ovulation.

**Methods:**

Cross-sectional study. The inclusion criteria were: patients with infertility for at least one year, complete examination and ovulation monitoring, aged between 18 and 38 years completed. The patients were divided into two paired groups: those who reported regular menstrual cycles and those who reported irregular cycles. In the ultrasonographic monitoring of ovulation we separated those who ovulated from those who did not ovulate, and applied the Fischer's test.

**Results:**

Among the 199 patients who reported having regular menstrual cycles, 113 had proven ovulation upon ultrasound monitoring and 86 patients did not ovulate. Among the 29 patients who reported irregular cycles, 24 did not ovulated at the cycle monitoring. The Fisher's exact test was applied and the p-value found was significant.

**Conclusion:**

The story of the patient in the clinical interview about the presence of regular or irregular menstruation correlates with the presence or absence of ovulation, it should be taken into consideration in the reasoning regarding the infertility etiology. This report would be important to guide the patient's ovulatory regularity diagnosis.

## INTRODUCTION

With female puberty, women reach fertility through the beginning of the monthly reproductive cycles that prepare the body for pregnancy (Moore, 2004). The menstrual cycle consists of all the physiological changes caused by the integrated action of the cortex-hypothalamus-pituitary-ovarian-uterine axis, and ends and begins with menstruation. The patterns of menstrual bleeding are considered relevant indicators of reproductive health, but few studies focus specifically on these aspects. Menstrual irregularities in the reproductive life of extremes are well known, however cycle variability in patients within reproductive age is not so clear. Bleeding patterns are hypothetically associated with hormonal fluctuations during the cycle. In the medical history of couple with infertility are the usual questions about the patient's menstrual regularity. Information is often based on the patient's memory, sometimes caught off guard by the question. The aim of this study is to assess whether the information on menstrual regularity is consistent with the assessment of the presence or absence of ovulation and if it is important as a component of the patient's clinical history.

### Review

#### Menstrual Cycle

The menstrual cycle can be divided into three phases: the follicular or proliferative stage, ovulation, and the luteal or secretory phase. The luteal phase has a fixed duration of 14 days, while the follicular phase can vary within a normal cycle. Ovulation is what defines a regular cycle. Both extra and intraovarian factors regulate folliculogenesis and there should be a balance between them (Artini *et al*., 2007). Any imbalance between the extra and intra ovarian factors may result in abnormal folliculogenesis (Frank *et al*. 2008). Among ovarian factors, one of the most, if not the most important, is the hypothalamus action, secreting the gonadotropin regulating hormone (GnRH) in pulses, which is transported to the anterior pituitary, where the cells produce, store, and secrete both the follicle-stimulating hormone (FSH) as well as the luteinizing hormone (LH). These hormones stimulate the ovaries to develop a dominant follicle, which secretes increasing levels of estrogen, which in turn cause endometrium proliferation. Estrogen inhibits the secretion of gonadotropins in the anterior pituitary and hypothalamus by indirect inhibition (negative feedback), and decreases the levels of LH and FSH. Above a certain level of estrogen, negative feedback is reversed and the release of LH from the pituitary gland is stimulated. This "LH peak" triggers ovulation. After ovulation, the remaining follicular cells in the ovary luteinize and form the corpus luteum, which secretes estrogen, but predominantly progesterone. This hormone, in turn, transforms the endometrium into a secretory and spongy tissue, necessary for the implantation of the fertilized ovum. If there is no implantation, human chorionic gonadotropin is not produced, and the corpus luteum regresses. Falling levels of estrogen and progesterone derivatives of the corpus luteum causes sloughing of the endometrium and thus menstruation.

Changes in the system can result in changes in ovulation, which will lead to changes in the menstrual cycle (Speroff & Fritz, 2005; Popat, 2008; Gray, 2013). This gonadotropin control process operates in the microenvironment of a follicle; however, the composition of the fluid mainly influences oocyte quality, and not so much the menstrual cycle itself (Chang *et al*., 2002; Salmassi *et al*., 2005).

#### Infertility and menstrual cycle

The United Nations define reproductive health as a complete physical, mental and social wellbeing, and not merely the absence of disease or infirmity in all matters relating to the reproductive system and to its functions and processes (United Nations, 1996). Infertility is a disease of the reproductive system, defined by the inability to achieve a clinical pregnancy after 12 months or more of regular, unprotected sexual intercourse (ASRM, 2013; Zegers-Hochschild *et al*., 2009; NICE, 2013). According to the American Society of Reproductive Medicine and the AHRQ (Agency for Healthcare Research and Quality) in the United States, because of the decline in fertility and increased time to conception after the age of 35, women aged above 35 years of age should be referred to the investigation of infertility after six months of trying to get pregnant without success (Liu & Case, 2011; ASRM, 2013). The WHO estimates that 48.5 million couples worldwide are infertile, and that between 1990 and 2010, the prevalence of primary and secondary infertility changed little in most of the planet (Mascarenhas *et al*., 2012). It is estimated that 7.4% of American couples are infertile, and in the world there is up to 15% of people with some fertility disorder, especially in industrialized nations (Sharma *et al*., 2013). Infertility should, therefore, be considered a disease process worthy of investigation and treatment. In the UK the principal causes of infertility factors are: men's factors (30%), ovulatory disorders (25%), tubal lesions (20%), uterus or peritoneal changes. Approximately 25% of infertility cases are of unknown etiology and in about 40% of cases, both partners had changes (NICE, 2013). The key features are regular menstrual cycles, frequency, intensity and duration of flow, but each of these characteristics shows considerable variability.

The nomenclature of menstrual disorders suffered a series of recommendations, eliminating Latin American terms of imprecise meaning, at the World Congress of Gynecology and Obstetrics of the International Federation of Gynecology and Obstetrics in Cape Town in 2009. If the interval between periods varies over 20 days, the cycle is considered irregular. As to frequency, the cycle is considered normal if the interval between the periods is between 24 and 38 days, infrequent menstrual bleeding being the name given to one or two bleeding episodes within a period of 90 days, and frequent menstrual bleeding represents more than four bleeding episodes during the same period in a given year. The duration of bleeding is considered normal if it is 4.5 to 8 days, and it is called abbreviated when it is below 4.5 days and extended if the duration is over 8 days. The normal volume is empirical and objectively perceived by the woman, being normal for a volume of 5 to 80ml of monthly blood loss, mild if less than 5 ml and accentuated above 80ml (Fraser *et al*., 2011). Table 1 summarizes these recommendations.

**Table 1:** Clinical dimensions of menstruation and menstrual cycle, and description of normal limits

	Denomination	References
Menstruation and menstrual cycle		
Monthly frequency (days)	Frequent	<24
	Normal	24–38
	Infrequent	>38
Regularity of menstruation Variation in 12 months (days)		
	Absent	
	Regular	2–20 days
	Irregular	>20 days
Duration of flow (days)	Prolonged	>8.0
	Normal	4.5–8.0
	Shortened	<4.5
Volume of monthly blood loss (mL)	Increased	>80
	Normal	5–80
	Light	<5

Fraser *et al*., 2011

## MATERIALS AND METHODS

### Type of study

Cross-sectional

### Local and study population

The study was carried out at the Human Reproduction Laboratory of the University Hospital, which serves patients of the Brazilian Public Healthcare System (SUS). The study was approved by the Ethics Committee of the University Hospital - Federal University of Goias. The following biochemical tests were requested for all patients: FSH between 2nd and 4th day of the cycle, LH, prolactin and thyroid-stimulating hormone (TSH). Ovulation monitoring was performed starting at the 2nd to the 5th day of the cycle, with reviews at the 10th day of the cycle until ovulation happened or until day 16, if there were no dominating follicles developing or follicular collapse. The ultrasound equipment used was a LOGIQ P6 model, manufactured by General Electrics (GE). The examinations were performed by doctors of the Human Reproduction Center HC-UFG.

### Design and variables

We evaluated 413 ultrasound monitoring reports of ovulation conducted between January 2011 and December 2015. The study included patients with infertility for at least one year, full examination of monitoring ovulation, aged between 18 and 38 years, no more than 10 mm follicles on 1st examination monitoring, the sum of the number of antral follicles between 6 and 24 between 2nd and 5th day of the cycle, absence of oophorectomy, no more than 40mm fibroids and/or submucosal and/or endometriosis tumors, FSH below 10UI/L, TSH between 0.5 and 4.5mU/L, and serum prolactin < 20mcg/L. We did not included patients diagnosed with polycystic ovary syndrome, smokers or those who reported drinking alcohol frequently.

**Figure 1:**
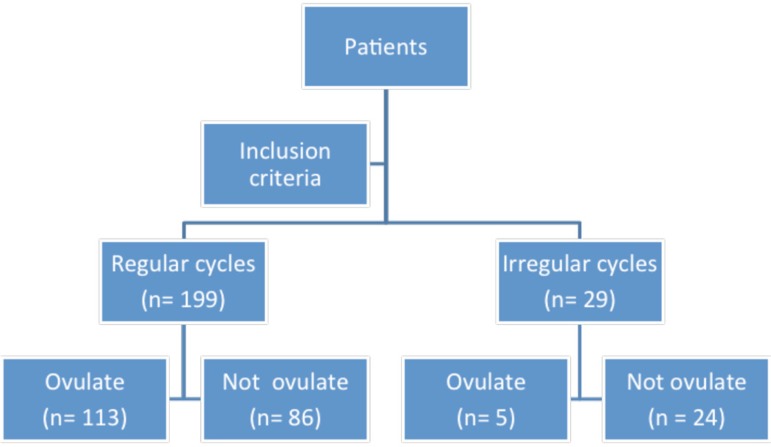
Study design

The patients were divided into two groups: those who reported having a regular cycle and those who reported irregularity of cycles. The definition of a regular cycle has been established in accordance with the International Federation of Gynecology and Obstetrics - FIGO (Table 1). The monitoring then identified those who ovulated and those who did not ovulate in the cycle in question (figure 1).

The presence of ovulation was found only in those cycles in which the follicle reached at least 16mm in diameter. The ovulation day calculation was performed according to the protocol suggested by Mikolajczyk *et al*. (2008). If the follicle reached 18mm or greater diameter, the ovulation day is considered as the day immediately following the largest follicle reaching a diameter before collapsing. In the case of follicular diameter between 16 and 18 mm, the second day after the examination before the collapse was considered as the day of ovulation. If the interval between these exams were more than five days, the patient was excluded from the study. A follicle collapse was considered upon the disappearance or decrease of at least 70% of its diameter.

To compare the groups of those who reported that they had a regular cycle and those who reported irregularity of cycles, they had to have similar characteristics so as to not influence the evaluation of the study variables. Both groups had to have a similar ovarian reserve, evaluated in this study by age, FSH, and the antral follicle count by ultrasound between the 2^nd^ and 5^th^ day of the cycle. Other factors can influence ovulation, such as thyroid function and serum prolactin levels. For this reason, both TSH values were matched as to the prolactinaemia between case and control groups. Although the menarche age, the number of pregnancies, births (parity), abortion, caesarean sections and Bilateral Tubal Ligation (BTL), do not have a direct relationship with the ovarian reserve or the possibility of ovulation during the ovulatory cycle, we consider it important to also pair these variables, to ensure group comparability. Samples of the groups were thus paired according to age, the age of first menstruation (menarche), the number of pregnancies, births, abortions and cesarean sections, the number of antral follicles counted between 2nd and 5th day of the cycle, BTL count, and the amount of laboratory tests of FSH (IU/L), prolactin (mcg/L) and TSH (mU/L) (Table 2). All inclusion criteria were based on the fact that they are factors that influence the menstrual cycle and fertility of women, and they may become confounders.

**Table 2:** Results from the t-test for unrelated samples, Mann-Whitney and Fisher’s exact test to assess the pairing of variables.

	t test (*P*)	Mann-Whitney (*P*)	Fisher (*P*)
Age (years)		0.745	
Menarche (years)		0.621	
Pregnancies		0.346	
Parity		0.844	
Abortions		0.191	
Caesarean		0.917	
Tubal sterilization			0.420
Antral follicle count		0.294	
FSH (UI/L)	0.425		
Prolactin (mcg/L)		0.957	
TSH (mU/L)		0.350	

*P*=0.05

### Statistical analysis

The paired data was submitted to the Shapiro-Wilk test, standard deviation calculation and value of Z to evaluate the type of data distribution: normal or not normal. The t test was used for normally distributed variables and the Mann-Whitney's test for non-normal distribution and Fischer's test for binomial variables. The test to evaluate the correlation between the cycle of regular reporting and presence or absence of ovulation, was the Fischer's test. The program used to perform the statistical tests was the SPSS 22.0.

## RESULTS

Only the variable with FSH values had a normal distribution curve (*P*> 0.05) according to the Shapiro-Wilk test. For this variable, there was the parametric t-test to assess the similarity between the groups. The other variables showed values with non-normal distribution (*P* <0.05), and submitted to the Mann-Whitney's test. The groups were subjected to statistical tests to see whether they were similar to the average of the variables desirable for the pairing. Therefore, all variables of interest were matched to ensure the homogeneity between the case and control groups.

There were 199 patients who reported regular cycles and 29 who reported irregular cycles. Among the 199 patients who reported having regular menstrual cycles, 113(57.78%) ovulated upon ultrasound monitoring and 86 (42.22%) patients did not. Among the 29 patients who reported having irregular cycles, 24 (82.76%) did not ovulate upon the cycle monitoring. Therefore, the patients who self-reported having irregular cycles had more than six times the likelihood of not ovulating in a menstrual cycle (Odds Ratio= 6.307). The chi-square Fisher test was found significant (*P* <0.0001) (Table 2).

## DISCUSSION

Menstruation can be conceptualized as the resulting periodic bleeding cycle (Deligeoroglou & Tsimaris, 2010). The ovarian cycle, because of the importance in steroidogenesis, and the endometrial cycle reflecting the end of the physiological changes of the shaft in the endometrium, name the phases of the menstrual cycle. Therefore, the proper functioning of the cortex, hypothalamic-pituitary-ovarian, endometrial axis is reflected on the regularity of menses and ovulation (Popat *et al*., 2008) and concludes in primary closing events for reproductive purposes: ovulation physiological endometrial changes and formation of the corpus luteum.

The fertility rate of women with long or irregular cycles is smaller, even in women with normal body mass index (Jensen *et al*., 1999). Infertility of ovulatory cause can reach up to a quarter of infertility etiologies and one of the questions in the clinical history of the patient is about the perception of the regularity of menstrual cycles. Evaluation of menstrual regularity, however, may not be a safe clinical parameter for the diagnosis of ovulation; there may be a lack of ovulation in women with regular menstrual cycles, and ovulation present in those with irregular menstrual cycles. In patients with amenorrhea, lack of ovulation is expected and widely known (Lauritsen *et al*, 2015). But many women cannot properly report on their cycle interval, days and amount of bleeding, stating only that the menstrual cycle is irregular. The assessment of ovulation through complementary examination such as ultrasound becomes a more accurate diagnosis of the presence of ovulation and the findings of Malcon & Cumming (2003) cast doubt on the concept of anovulatory cycles in eumenorrheic women (Malcolm & Cumming, 2003). Eumenorrheic women may have anovulatory cycles, like so, there is no absolute relationship between menstrual regularity and ovulation, and the anovulation may be a subclinical cycle dysfunction (Hambridge *et al*., 2013). Carmina & Lobo (1999) observed 20 to 50% of anovulatory cycles in patients with self-reported regular cycles, but also observed that patients with prolonged interval between cycles do not necessarily have anovulation.

Another motivation for this study was to strengthen, together with current data, the need to take a detailed medical history, important for the initial investigation of infertility by the clinician. With technological advances, the clinical history often goes into the background, and the general gynecologist feels inhibited and uncertain about the importance of the anamnesis.

In our study, the patients who reported having regular menstrual cycles, correlated with ovulation monitoring showed the presence of ovulation, and who reported irregular menstrual cycles showed correlation with lack of ovulation on ultrasound scans. In the absence of other diseases, the report that the menstrual cycle is irregular, can facilitate and even drive the reasoning for the diagnosis of anovulation, given the significance of the statistical results.

## CONCLUSION

The patient's information in the clinical interview about the presence of regular or irregular menstruation correlates with the presence or absence of ovulation. It is a question that should continue to be asked when collecting information from the clinical history, and should be valued when looking for the etiology of infertility. This report would be important to guide the diagnosis of ovulatory regularity of the patient.

## Figures and Tables

**Table 3:** Association between the cycle regularity report and presence or absence of ovulation.

	n	Irregular	Regular	*P*-value	OR	CI (95%)
						
Not ovulated	110	24	86	<0.0001	6.307	2.3121-17.2046
Ovulated	118	5	113			

OR= Odds Ratio; CI = Confidence Interval.
